# Recovery of photoacoustic images based on accurate ultrasound positioning

**DOI:** 10.1186/s42492-021-00072-2

**Published:** 2021-03-25

**Authors:** Yinhao Pan, Ningbo Chen, Liangjian Liu, Chengbo Liu, Zhiqiang Xu, Jianhui Zhang

**Affiliations:** 1 College of Mechanical and Electrical Engineering, Guangzhou University, Guangzhou, 510006 China; 2 Research Laboratory for Biomedical Optics and Molecular Imaging, CAS Key Laboratory of Health Informatics, Shenzhen Institutes of Advanced Technology, Chinese Academy of Sciences, Shenzhen, 518055 China

**Keywords:** Photoacoustic microscopy, Polygon-scanning, Image correction, Ultrasound positioning

## Abstract

Photoacoustic microscopy is an in vivo imaging technology based on the photoacoustic effect. It is widely used in various biomedical studies because it can provide high-resolution images while being label-free, safe, and harmless to biological tissue. Polygon-scanning is an effective scanning method in photoacoustic microscopy that can realize fast imaging of biological tissue with a large field of view. However, in polygon-scanning, fluctuations of the rotating motor speed and the geometric error of the rotating mirror cause image distortions, which seriously affect the photoacoustic-microscopy imaging quality. To improve the image quality of photoacoustic microscopy using polygon-scanning, an image correction method is proposed based on accurate ultrasound positioning. In this method, the photoacoustic and ultrasound imaging data of the sample are simultaneously obtained, and the angle information of each mirror used in the polygon-scanning is extracted from the ultrasonic data to correct the photoacoustic images. Experimental results show that the proposed method can significantly reduce image distortions in photoacoustic microscopy, with the image dislocation offset decreasing from 24.774 to 10.365 μm.

## Introduction

Photoacoustic imaging (PAI) is a biomedical imaging technology that has rapidly developed in recent years. PAI is based on the photoacoustic effect; that is, after matter-absorbed radiant energy or pulsed light is modulated, part of the light energy is converted into thermal energy. The periodic thermal expansion and cold contraction of substances produce ultrasonic waves via thermoelastic effects. Photoacoustic microscopy (PAM) is an important branch of photoacoustic imaging. PAM has a high sensitivity to light absorption, which enables the study of multiscale, multi-parameter, and multi-contrast biological systems [1, 2]. PAM is a focused scanning imaging technology in which a focused laser is used to excite a photoacoustic signal in the sample. Ionized water and ultrasonic glue are used as the photoacoustic coupling medium between the sample and probe. The excitation optical path is coaxially aligned with the focus of the ultrasonic transducer to detect photoacoustic signals with high sensitivity. Two-dimensional scanning along the tissue surface is used to reconstruct the three-dimensional tissue information.

Traditional PAM systems employ a linear motorized stage for point-by-point scanning [3]. Traditional scanning has high displacement accuracy and a large imaging range; however, the scanning speed is considerably limited because of the large volume of the linear translation stage. Microelectromechanical system (MEMS) scanning mirrors [4] can be used to increase imaging speed. However, only the laser beam is scanned in MEMS systems, which makes it difficult to realize the confocal alignment of the incident laser and the ultrasonic transducer. Consequently, the system has a low signal-to-noise ratio (SNR), and the imaging range is limited by the focusing range of the ultrasonic detector. To solve this problem, a MEMS scanning mirror was designed that can be immersed in water to achieve simultaneous scanning of laser and ultrasonic signals. This technology thereby maintains confocal alignment of the incident laser and the ultrasonic transducer. However, the scanning range of MEMS mirrors depends on the driving frequency; that is, the scanning range is largest when the driving frequency is close to the resonant frequency, which affects the imaging range at different scanning rates. As the driving frequency of the MEMS scanning mirrors depends on the mirror size, a very small mirror must be used when a high scanning frequency is required. This results in the low reflection efficiency of the MEMS mirror for laser and ultrasonic signals, and it affects the image quality [4–8].

In recent years, polygon-scanning has been proposed, whereby a polygon mirror (consisting of six rotating mirrors, for example) can be immersed in a water tank, and the polygon mirrors are rotated by a motor to rapidly and simultaneously scan the incident laser and photoacoustic signals. Accordingly, confocal detection is achieved. The installation and manufacturing of polygon mirrors introduce geometric errors into the surface flatness, tilt angle, and distance from the polygon-scanning axis to each mirror. Thus, random fluctuations occur in the rotating speed during the scanning process of the polygon mirrors, and random jitter occurs in the spatial position of the polygon scan. All these factors result in errors in the scanning positions of the six mirrors, which in turn leads to dislocations between the adjacent B-scan images of the reconstructed photoacoustic microscopic images [5, 9].

To resolve these problems, an image recovery method is herein proposed for photoacoustic microscopy systems using polygon-scanning. The proposed method is realized in two main parts: first, the photoacoustic and ultrasonic imaging data of the sample are obtained during imaging scanning. Then, the angle information of each mirror of the polygon mirrors is extracted from the ultrasonic data to correct the photoacoustic images in the data processing part. During imaging, the system first emits an ultrasonic wave and then a laser after a delay of a few microseconds. Through an ultrasonic transducer, the system receives a photoacoustic signal and then finally receives an ultrasonic signal. This process collects the photoacoustic signal of a single imaging point and obtains the rotation angle of the rotating mirror at that time. In addition, although the ultrasonic and laser beams are not excited simultaneously, the rotating speed of the rotating mirror is much slower than the delay time between the ultrasonic and laser beams. It is therefore contended that both the ultrasound signal and photoacoustic signal are detected from the same point.

## Methods

### Polygon-scanning system

**Fig. 1:**
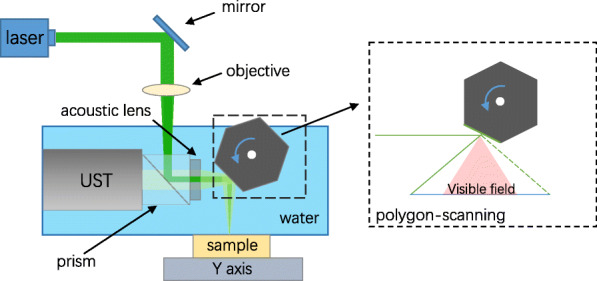
System schematic. A laser at a 532 nm wavelength is coaxially coupled with an ultrasound beam through a photoacoustic prism and is reflected onto the sample by a rotating mirror. With the rotation of the rotating mirror, the position of the optical beam and sound beam reflected on the sample will change. The scanning area is divided into the visible field and invisible field

The imaging system is shown in Fig. 1. A Nd:YAG pulsed laser (532 nm GKNQLML 532–6-10, China) is focused using a mirror (GMH11-025A, Hengyang), an objective lens (ACN254-050A, Thorlabs), and an aluminized coupling prism. A polygon mirror is employed to reflect the laser that is focused on the sample. An ultrasonic transducer (UST, V214-BB-RM, Olympus NDT) transmits the ultrasonic signal and receives both the photoacoustic signal produced by the tissue and the ultrasonic signal reflected by the tissue. The ultrasound is aligned to be confocal with the optical beam through a prism and an acoustic lens [6] (#63–477, Edmund). The acoustic lens is closely connected to the photoacoustic prism through optical adhesive technology. The polygon scanning mirror contains six identical mirrors. As the polygon mirror rotates clockwise, the laser and ultrasound beam are reflected on the sample by the six rotating mirrors, thereby realizing rapid scanning in one direction, which is defined as the fast axis. The fast axis is perpendicular to the linear translation stage (VT-802SM, Physik Instrumente) on which the sample is placed. The motorized stage moves along the slow axis during the polygon mirror scanning along the fast axis, thereby expanding the system scanning dimension to realize a two-dimensional scan. Figure 1 shows that increasing the angle of the rotating mirror increases the distance traveled by light and sound to the sample. When this distance is too large, the light and sound will be out of focus, and useful signals cannot be obtained. This region is called the invisible area, and its counterpart is called the visible area [3, 10].

**Fig. 2:**
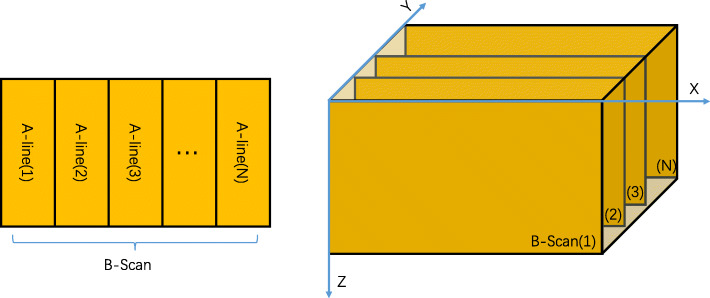
(**a**) B-scan data structure and (**b**) overall data structure

Figure 2 shows the data structure of the raw image data generated by the system during imaging. Each photoacoustic signal from the detected point is called an A-line, which contains the information of the tissue in the Z direction. The X direction is called the B-scan direction, and the Y direction is called the slow-axis scanning direction. Using the maximum value of the A-line to perform a projection along the Z direction yields the imaging result, which is called the maximum amplitude projection (MAP). The cross-sections in the X-Z plane are B-scans [11].

**Fig. 3:**
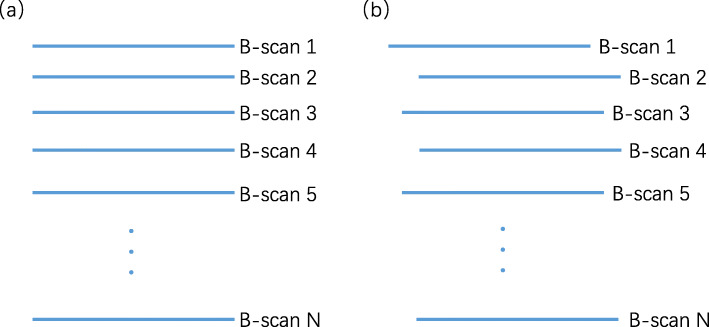
MAP obtained under (**a**) ideal conditions and (**b**) actual conditions

Ideally, the obtained MAP should be dislocation-free, as shown in Fig. 3a. However, the actual MAP contains a dislocation, as depicted in Fig. 3b. Analysis shows that the dislocation is mainly produced because of the fluctuation of the motor speed during the rotation of the polygon mirror, which makes the cycle of each system B-scan inconsistent. That is, each mirror will reach the same specific angle in a different time period. Splicing the image reconstruction using the same B-scan period produces a clearly misplaced image. The rotation (angle) information of the polygon-scanning can be used to determine the time required for each B-scan to guide the stitching of the image reconstruction. Consequently, the ultrasound image can be used to obtain the polygon-scanning rotation (angle) information to guide the image stitching reconstruction.

### Ultrasonic acquisition of angle information

**Fig. 4:**
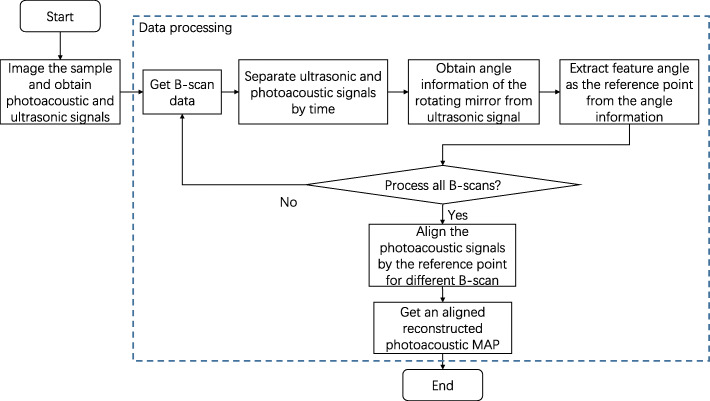
Flow chart of the proposed method

**Fig. 5:**
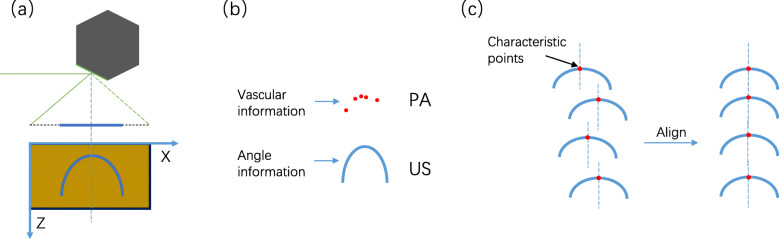
Method of splicing photoacoustic signals according to the rotation angle information. **a** Relationship between the angle information for polygon-scanning and B-scan image. **b** Relationship between the photoacoustic signal and ultrasonic signal in the B-scan image. **c** Reference alignment of each B-scan using reference points

A flowchart of the proposed method is presented in Fig. 4. This method first scans the sample as a whole. After scanning, photoacoustic and ultrasonic signals are obtained. The two signals are distinguished based on the difference in the flight time of the ultrasound signal. The PA signal is the information of the sample, while the ultrasonic signal is the angle information of the rotating mirror. In each B-scan, the same characteristic angle contained in the ultrasonic signal is extracted as the reference point. The reference point is used as the alignment point between different B-scans of the PA signal for splicing and reconstruction. Finally, a photoacoustic MAP with low dislocation is obtained. A change in the angle of the rotating mirror will cause a change in the distance from the detection point of the sample to the ultrasound transducer. The rotation information (rotation angle) of the polygon-scanning can be obtained from the time at which the signal arrives at the transducer, as shown in Fig. 5a. However, the photoacoustic signal detected from the tissue relies on a discretely distributed vascular network. Therefore, it cannot obtain the complete angle information of polygon-scanning. The difference is that the ultrasonic signal receives the ultrasonic echo reflected from the surface of the sample and does not depend on the vascular network. It is thus more reliable to obtain the angle information through ultrasound [3]. Consequently, ultrasound images from the sample are used to detect the polygon-scanning angle information. The angle information obtained by ultrasound is used to guide the reconstruction and stitching of the photoacoustic image; that is, photoacoustic and ultrasonic imaging are simultaneously performed, photoacoustic imaging is employed to obtain the sample biological information, and ultrasonic imaging is used to obtain the angle information for polygon-scanning, as shown in Fig. 5b. The ultrasonic B-scan image is arc-shaped, and the points of the arc correspond to the angles of the rotating mirror. The vertex of the arc is taken as the characteristic point of each B-scan, where the angle of the rotating mirror makes the ultrasound vertically incident on the sample surface. At this characteristic point, the ultrasound takes the shortest path to reach the sample. The characteristic angle is used to align and stitch all the B-scans to eliminate the inconsistency in the time period of each B-scan from the fluctuating motor speed. This prevents dislocations between adjacent B-scans and improves the image quality (see Fig. 5c).

## Experiments

**Fig. 6:**
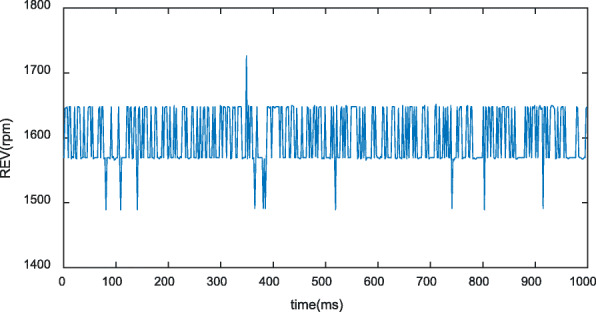
Motor speed. The motor speed is set at 1600 rpm, and the actual real-time speed of the motor fluctuates near 1600 rpm. After the deceleration ratio of 16:1, the average speed of the rotating mirror is 100 rpm

**Fig. 7:**
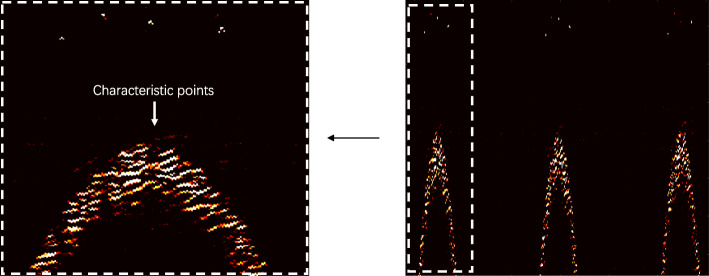
Actual photoacoustic and ultrasonic signals under B-scan. The upper part is the photoacoustic signal, the lower part is divided into ultrasonic signals, and the highest point of the ultrasonic signal is set as the characteristic point

**Fig. 8:**
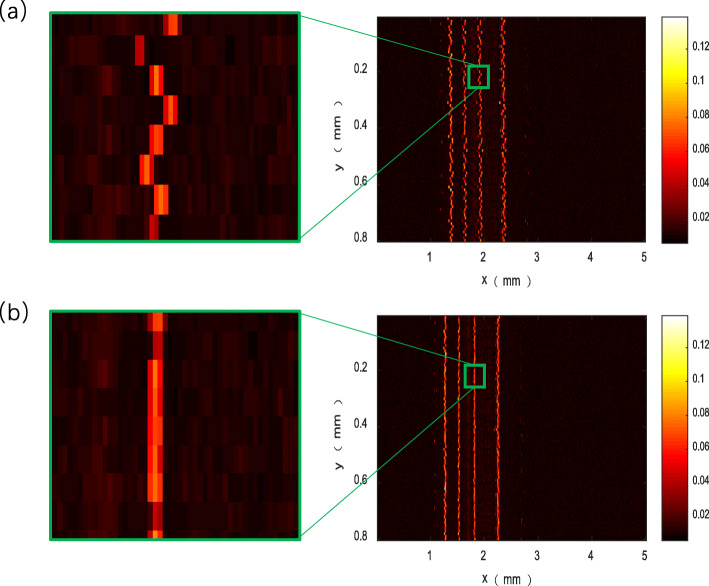
Multiple B-scans of the same area of four tungsten wires with a diameter of 40 μm. Photoacoustic MAP images of the tungsten wire (**a**) before correction and (**b**) after correction

The objective lens used in the experiment had a numerical aperture (NA) of 0.025, which resulted in a lateral resolution for the system that could be determined using the formula ${\boldsymbol{R}}_{\boldsymbol{L}}=\mathbf{0.51}\frac{\ \boldsymbol{\uplambda}}{\boldsymbol{NA}}=\mathbf{10.85}\boldsymbol{um}$ [1]. The laser repetition frequency was 10 kHz. It was ensured that the scanning step of the fast axis was within the aforementioned resolution by setting the average speed of the motor to 1600 rpm/min and the deceleration ratio of the gearbox to 16:1. Because there were six B-scans in each rotation cycle during the scanning, the B-scan rate was calculated to be 10 Hz. The scanning range was 10 mm, such that the scanning step in the B-scan direction was 10 μm. The velocity of sound in water is Vs = 1500 μm/s, the frequency of the ultrasonic transducer is 50 MHz, and the axial resolution of the system ultrasound was calculated to be 27 μm using the formula ***R***_***A***_ ***=*** **0.88*V***_***s***_/***f*** [1]. The system was used for photoacoustic and ultrasonic scanning of imitation tungsten wire. To avoid introducing a sample error, we scanned the sample at the same position many times and spliced the scanned data to reconstruct the image. The built-in encoder of the rotating motor for polygon-scanning was used to monitor fluctuations in the motor speed during the scanning process, as shown in Fig. 6. The data show that the motor speed fluctuates around 1600 rpm/min, with a maximum fluctuation of 126.4000 rpm/min. The collected tungsten wire data were spliced and reconstructed. Simultaneously, the ultrasonic signal was used to identify the feature points of each B-scan, which were used for correction and reconstruction, as shown in Fig. 7. The reconstruction result is shown in Fig. 8. This result can be effectively used to obtain angle information for polygon-scanning, which can then be used to guide photoacoustic image stitching and reconstruction.

### Quantitative analysis

**Fig. 9:**
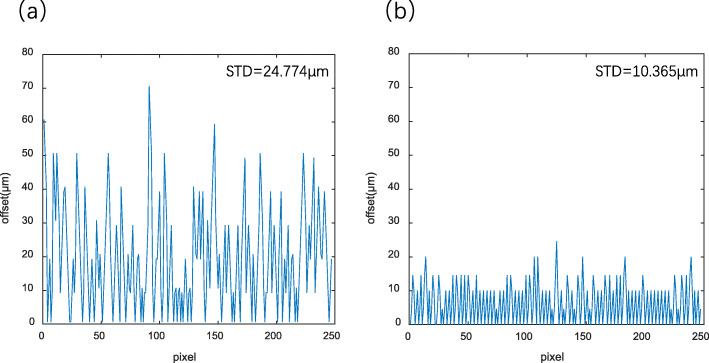
Position offset of the x-coordinate position of each pixel. The offset is calculated using the absolute value. **a** Before correction and (**b**) after correction

Quantitative analysis of one of the tungsten wires shown in Fig. 7 was conducted to demonstrate the position offset of each pixel in the x-coordinate of the tungsten wire before and after correction, as shown in Fig. 9. The step size in the x-direction is 10 μm/step. The standard deviation of the position offset for Figs. 7a and 7b is calculated as 24.774 and 10.365 μm, respectively. Thus, the correction method can reduce the number of image dislocations to less than half. The effect caused by the fluctuation of the motor speed is eliminated; however, the dislocation in the image remains. This is caused by the difference in the scanning range of different B-scans resulting from manufacturing and assembly errors of the polygon mirror. It is also due to other factors, such as the inconsistency of the distance from the surface of each mirror to the center of the polygon, and the eccentricity of the polygon mirror caused by the mismatch between the centerlines of the polygon mirror and the motor shaft. Thus, improving the manufacturing and assembly accuracy of polygon mirrors can reduce image dislocation.

## Conclusions

Polygon-scanning is used to guide the stitching and reconstruction of photoacoustic images. In this paper, we proposed a method for using ultrasound to obtain angle information from polygon-scanning, thereby effectively reducing dislocations in spliced reconstructed images and improving the image quality. The effectiveness of the proposed method was verified by performing experiments on a tungsten wire. Quantitative analysis showed that the proposed method reduced the dislocation of polygon-scanning imaging from 24.744 to 10.365 μm.

## Data Availability

All the functional modules will be shared on GitHub.

## Abbreviations

PAI: Photoacoustic imaging
PAM: Photoacoustic microscopy
MEMS: Microelectromechanical systems
UST: Ultrasonic transducer
PA: Photoacoustic signal
US: Ultrasonic signal
MAP: Max amplitude projection
NA: Numerical aperture
